# Novel Pinhole Emitter Chip for Micro Supercritical Fluid Chromatography–Mass Spectrometry with Integrated Dilution-Free Fluidic Back-Pressure Regulation

**DOI:** 10.1021/acs.analchem.4c05171

**Published:** 2024-12-02

**Authors:** Julius Schwieger, Chris Weise, Detlev Belder

**Affiliations:** Institute of Analytical Chemistry, Leipzig University, Linnéstraße 3, 04103 Leipzig, Germany

## Abstract

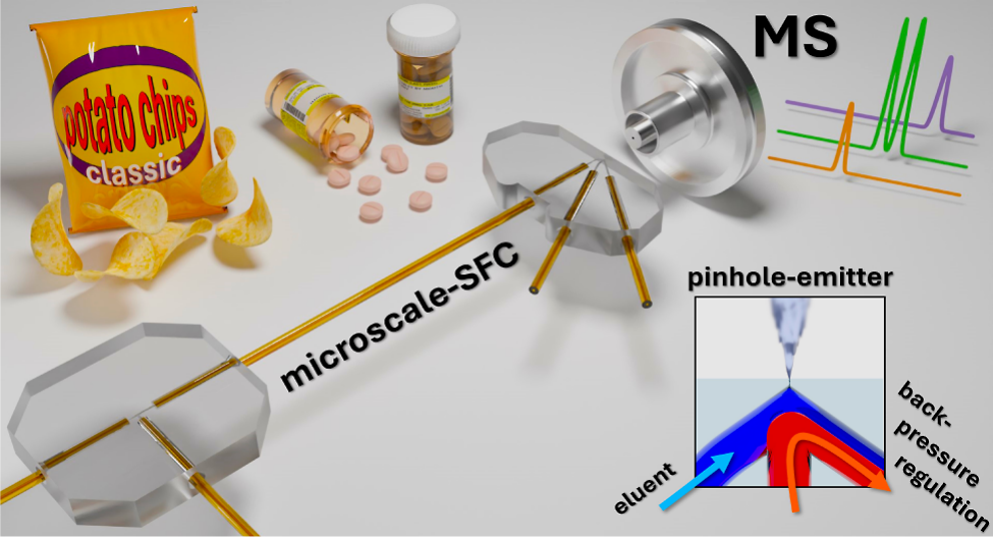

We present a novel chip-based device featuring a pinhole emitter for mass spectrometry (MS) coupling with integrated fluidic back-pressure regulation for supercritical mobile phases. This design enables facile coupling of packed capillary columns used for supercritical fluid chromatography (SFC) with atmospheric pressure ionization mass spectrometry. The monolithic microfluidic chips were fabricated using selective laser-induced etching, seamlessly integrating multiple functions, including comb-shaped particle retention structures for column packing and ports for zero-clearance connection with standard fused silica capillaries. The integrated restrictive pinhole MS emitter generated by dielectric breakdown is a key innovation of the micro SFC–MS platform. It enables a controlled decompression of the supercritical CO_2_-based mobile phase within few micrometers to efficiently transfer the analytes from the compressed supercritical fluid into the ambient gas phase in front of the MS orifice. The inclusion of an arrowhead-shaped fluidic element further enables precise, dilution-free back-pressure regulation. With a minimal postcolumn volume of just 3 nL, the system shows excellent MS coupling performance, as demonstrated by rapid SFC–MS analysis of pharmaceuticals and natural products.

## Introduction

Driven by the demand for higher sample throughput and a growing emphasis on sustainability, supercritical fluid chromatography (SFC) has emerged as a promising technology in analytical chemistry.^1−4^ Despite the early discovery of SFC in 1962,^5^ it had long suffered from declining interest due to overly optimistic expectations—a common fate for cutting-edge innovations.^6,7^ However, recent breakthroughs in instrument development have turned SFC, once derided as “science fiction chromatography”, into practical reality.^8,9^ The use of supercritical carbon dioxide (scCO_2_) as a mobile phase combined with organic modifiers has enabled chromatographic separations within seconds^10^ as well as the simultaneous analysis of both nonpolar and polar substances within a single analytical run.^11^ The essential characteristics of scCO_2_ (*p*_c_ = 74 bar, *T*_c_ = 31 °C) that facilitate such advancements are low viscosity and fast mass transfer kinetics. These advantages have become so significant that even SFC’s main competitor, high-performance liquid chromatography (HPLC), aims to replicate them by employing high-temperature methods.^12−14^ However, not only have the instrumental advancements in SFC brought the technology into the spotlight, but also its various upstream and downstream hyphenation capabilities.^15,16^ In this regard, supercritical fluid extraction (SFE) stands out in particular due to the significant overlap in the instrumentation required. The combination of SFE and SFC enables semipreparative separations, making it an ideal choice for the analysis of natural products.^17^ The use of scCO_2_ as both extractant and mobile phase simplifies sample preconcentration through decompression, streamlining the process and enabling high-throughput methods.^17−19^ Back-end hyphenation for detection primarily relies on mass spectrometry (MS), which is usually the most information-rich detection technique.^20^ When coupling SFC with ambient pressure ionization MS instruments, the use of scCO_2_ promotes the efficient nebulization of the mobile phase through decompression.^21^ Although MS interfacing becomes substantially more complex due to the compressibility of scCO_2_ and the requirement for precise pressure regulation, this approach enables superior detection sensitivity.^22,23^

As the unique properties of scCO_2_ are best utilized by combining SFC with complementary techniques, the development of fully integrated systems is a strategic next step. This would allow the combination of chemical reaction, extraction, separation, and detection on a single platform while significantly reducing instrumental complexity. Previous studies have highlighted that chip-based microfluidics offer a promising approach to drive such developments forward.^24,25^ This awareness is also reflected in the increasing research aimed at developing corresponding microscale SFC functionalities, such as back-pressure regulators,^26^ temperature sensors,^27^ flow regulators,^28^ extraction devices,^29,30^ and microreactors.^31^

However, despite the advanced technical maturity and commercial availability of chip HPLC–MS,^32,33^ the development of chip SFC–MS remains challenging. This is mainly due to the compressible nature of scCO_2_. As the viscosity and mass flow of the mobile phase along the column are strongly pressure-dependent, adjustable back-pressure regulators (BPRs) are required. These regulators facilitate the optimization of separation parameters and maintain reproducibility by pressure stabilization. Since commercial BPRs are unsuitable for microfluidics due to their comparatively large swept volumes, the implementation of miniaturized alternatives represents a critical challenge. This issue is even more pronounced in SFC–MS interfacing, where the mobile phase must transition from a pressurized supercritical to an ambient gaseous state without losing chromatographic integrity.^34^ This limitation has been frequently discussed in recent literature but lacks a definitive solution.^35−37^ The most commonly used approach in classical macroscopic SFC–MS equipment is the pressure-regulating fluid interface. It consists of a makeup pump, a BPR-controlled splitter, and an emitter-capillary.^37,38^ However, this very practical solution has disadvantages regarding sample dilution and hinders further miniaturization due to large void volumes.^35^

We recently reported a microscale pressure-regulating fluid interface as part of a modular miniaturized SFC–MS platform.^39^ By combining the benefits of capillary and chip-based microfluidics, this approach enabled straightforward capillary SFC–MS coupling with record-speed chiral SFC–MS analysis. However, avoiding sample dilution was challenging with the X-shaped microfluidic makeup flow-BPR approach, especially at varying flow conditions. The same applies to controlling the defined expansion of the mobile phase due to the relatively long capillary, which must function as both a restrictor and an emitter.

These issues can be circumvented with the novel chip-based device presented in this contribution, which features a pinhole emitter instead of an adjacent capillary for MS coupling and a novel arrowhead-shaped structure that seamlessly integrates fluidic back-pressure regulation without analyte dilution.

## Experimental Section

### Chemicals and Materials

The solvents methanol, isopropanol, acetonitrile and ethanol (HPLC grade) were purchased from VWR International LLC (Radnor, PA, USA). Pressurized CO_2_ (purity grade N45) for the SFC pump was obtained from Air Liquide (Paris, France). Formic acid (LiChropur, 98–100% purity), disodium hydrogen phosphate as well as rhodamine 6G (98%), warfarin (PESTANAL, analytical standard), (±)-α-tocopherol (95.5%), (±)-β-tocopherol (CERILLANT, reference material), (+)-γ-tocopherol (CERILLANT, reference material), (+)-δ-tocopherol (SUPELCO, analytical standard) and 6-hydroxyflavanone were obtained from Sigma-Aldrich (Taufkirchen, Germany). Mianserin hydrochloride (98%) was obtained from abcr GmbH (Karlsruhe, Germany). NH_3_ (25%) was purchased from Carl Roth GmbH (Karlsruhe, Germany). The chiral stationary phases IG-3 and IA-3 (fully porous, *d*_p_ = 3 μm) were provided by Daicel (Osaka, Japan). The stationary silica phases ProntoPEARL 120-2.2-Si (fully porous, *d*_p_ = 2.2 μm) and Exil Pure 120 (fully porous, *d*_p_ = 3 μm) were provided by BISCHOFF Analysentechnik und Geräte GmbH (Leonberg, Germany) and Dr. Maisch (Ammerbuch-Entringen, Germany). Connecting parts for the microfluidic setup and tubings were obtained from both VICI AG International (Schenkon, Switzerland) and IDEX Health & Science (Oak Harbor, WA, USA).

### Sample Preparation

Potato chips “Crunchips-African Style” from Lorenz Bahlsen Snack-World GmbH & Co KG (Neu-Isenburg, Germany) were purchased in 2023 from a local supermarket in Leipzig, Germany. A sample preparation method was applied that was originally developed by Martínez-Vidal et al. for olive oil and further improved by Gu et al. for the analysis of sterols and tocopherols via liquid chromatography–MS.^40,41^

The potato chips were ground, and a small sample (1.0 g) was transferred into a 10 mL round-bottom flask. It was wrapped in aluminum foil, equipped with a Dimroth condenser and put into a water bath. 5.0 mL of *n*-hexane was added and stirred for 1 h at 60 °C. The turbid solution was then centrifugated (10 min, 2500*g*), and 3.0 mL of the supernatant was returned into the flask. 2.5 mL of ethanolic KOH solution (2 M) and 0.5 mL of an aqueous l-ascorbic acid sodium solution (0.2 g/mL) were added and stirred for 90 min at 60 °C. The solution was left to cool at room temperature and washed four times with water (3.0 mL) until the washing solution gave a neutral reaction. The extract was then dried with anhydrous sodium sulfate. The clear yellow solution (1.0 mL) was stored at −20 °C and used for SFC–MS analysis without further dilution.

### Chip Layout and Fabrication using Selective Laser-Induced Etching

The microfluidic glass chips were fabricated using selective laser-induced etching (SLE), a process consisting of two sequential steps.^42^ First, the substrate is selectively modified using a femtosecond laser, followed by chemical etching to remove the targeted regions. The structures to be incorporated into the glass were designed first as computer-aided design (CAD) models generated with Autodesk Inventor Professional 2024 (San Rafael, CA, USA). The dimensions of the CAD-models were calculated considering the impact of the etching procedure with etching rates of 1 μm/h for pristine and 230 μm/h for structured fused silica to provide spatial accuracy. Channels and inlets had to be designed separately from the μ-frit, as they were processed and etched individually during fabrication. The sequential process is necessary since small structures (>10 μm) would overly enlarge by the time necessary to dissolve larger structures. The CAD models were exported as .stp files and transferred to the computer-aided manufacturing (CAM) software Alphacam 2017 R2 (Vero Software GmbH, Neu-Isenburg, Germany). Alignment crosses were added to match the individually processed large and fine structures. The CAM file was then converted into machine code and transferred to the SLE device (FEMTOprint aHead P2, FEMTOprint SA, Muzzano, Switzerland). First, the channels and inlets were processed on a fused silica wafer using a pulsed femtosecond IR laser. Subsequently, the laser-treated substrate was washed with deionised (DI) water and submerged into a hot etching solution inside a pulsing ultrasonic bath (2 min on, 13 min off) for 20 h. The substrate was then removed and thoroughly cleaned by sonicating for 30 min with DI water. The dried microstructured wafer was reintroduced into the SLE device for structuring the μ-frits. After aligning, the fine structures were processed, followed by a shortened etching procedure of 2 h 15 min. It was sonicated continuously for the first hour to ensure the reentry of the etching solution inside the microfluidic structures. Simultaneously, the outlines of the chips were etched, causing the chips to be separated from the wafer. The chips were then washed with DI water, submersed into a casting solvent (6:2:2 acetonitrile, ethanol, aqueous 5 mM phosphate buffer) for 15 min and dried on a heating plate at 90 °C. A more comprehensible overview of the individual steps of the SLE-fabrication process, including the processing parameters, is given in Figure S1 in the Supporting Information.

For the chip-based SFC–MS platform, two chip modules were fabricated: a tee-junction chip featuring a μ-frit and a flow-split structure and an emitter chip, which includes a μ-frit along with an integrated emitter and a microfluidic BPR structure. The connection between the chips and the fluidic periphery was achieved via cylindrical cavities (inlet structures) inside the glass that allowed for the introduction of fused silica capillaries with an outer diameter of 360 μm. Length and inner diameter (ID) of the capillaries were chosen according to their individual function and position: The emitter chip was equipped with a column capillary (11 cm, ID 100 μm) adjacent to the frit structure, the inflow for the MeOH (6 cm, ID 100 μm) and the outflow to the BPR (6 cm, ID 100 μm). The tee-junction chip was equipped with a mobile phase inflow at the tapered channel (6 cm, ID 50 μm) and an adjacent split-outflow to the BPR (6 cm, ID 100 μm). After the emitter and column manufacturing, the vacant inlet at the frit structure is connected to the column capillary (Figure S2). All capillaries were prepared by cutting the raw material to the corresponding length using a Shortix capillary GC column cutter from Scientific Glass Technology (Middelburg, Netherlands). The cutting surfaces were polished flat using a capillary polishing station (MS Wil, Aarle-Rixtel, Netherlands). Grinding residues were flushed out with isopropanol, and the remaining liquid evaporated on a heating plate at 100 °C. The capillaries were glued into the corresponding vacancies using the epoxy adhesive ClearWeld from JB Weld (Sulphur Springs, TX, USA). Prior to use, the glue was allowed to cure for 5 min to reach sufficient viscosity.

### Pinhole Emitter and Column Manufacturing

On the emitter chip, an 8–15 μm long crack between the sharp V-shaped channel joint and the edge of the glass served as a restrictive emitter structure for the controlled decompression of scCO_2_ and the transfer of analytes into the gaseous phase. This “nanochannel” was generated by inducing an electric breakdown through the slim glass wall. The manufacturing procedure was adapted from Mao et al.^43^ and is further described in Figure S2.

The column manufacturing was conducted using a pump-driven slurry packing approach, similar to the procedure presented in more recent work.^39^ Briefly, the capillary column attached to the emitter chip was connected to a packing cartridge filled with slurry (1–2 mg/mL IG-3, IA-3 in acetonitrile or 1–2 mg/mL silica 2.2/3.0 μm particles in MeOH). The particles were flushed toward the chip using an HPLC pump (LC-20AD from Shimadzu, Kyoto, Japan) at constant pressure mode (350 bar) and retained at the SLE-fabricated μ-frit on the emitter chip. After reaching the desired column length, the system was depressurized. The column was stored upright overnight for the particles to sediment and the solvent to evaporate. The capillary column was cut to the desired length and glued into the tee-junction chip. A comprehensive depiction of the entire assembly of the microscale SFC system is given in Figure S2 in the Supporting Information.

### SFC and MS Instrumentation

Analyte separations were carried out by connecting the chip-based SFC platform to a 1260 Infinity SFC System from Agilent Technologies (Santa Clara, CA, USA). The integrated binary pump of the system provided the mobile phase as an adjustable mixture of scCO_2_ and modifier (MeOH, 0.1% FA or 1:1 MeOH/*i*PrOH, 0.1% NH_3_). Since the employed SFC system is pressure controlled, the preset flow of the mobile phase (1.0–1.5 mL/min) was split toward a heated BPR (50 °C) before entering the chip-based platform to keep the desired pressure value in the precolumn area.^44^

Analyte ions were detected with the single quadrupole mass spectrometer 6150B from Agilent Technologies (Santa Clara, CA, USA) operating in both polarities. The emitter chip was placed near the MS capillary (2–3 mm distance) with the spray shield and the capillary cap removed. Gas flow (N_2_) and dry gas temperature were set to 1 L/min and 180 °C (pos. mode) or 250 °C (neg. mode). The MS inlet voltage was set to −4.5 kV for positive mode and 3.0 kV for negative mode if not stated otherwise. Analyte ion chromatograms were obtained using selected ion monitoring (SIM): warfarin ([M + H]^+^ = 309.4 *m*/*z*, fragmentor voltage 250 V), mianserin ([M + H]^+^ = 265.4 *m*/*z*, 150 V), 6-hydroxyflavanone ([M + H]^+^ = 370.4 *m*/*z*, 150 V), α-, β-, γ-, δ-tocopherol ([M + H]^+^ = 431.4 *m*/*z* for α, [M + H]^+^ = 417.4 *m*/*z* for β and γ, [M + H]^+^ = 403.4 *m*/*z* for δ, 250 V each).

**Figure 1:**
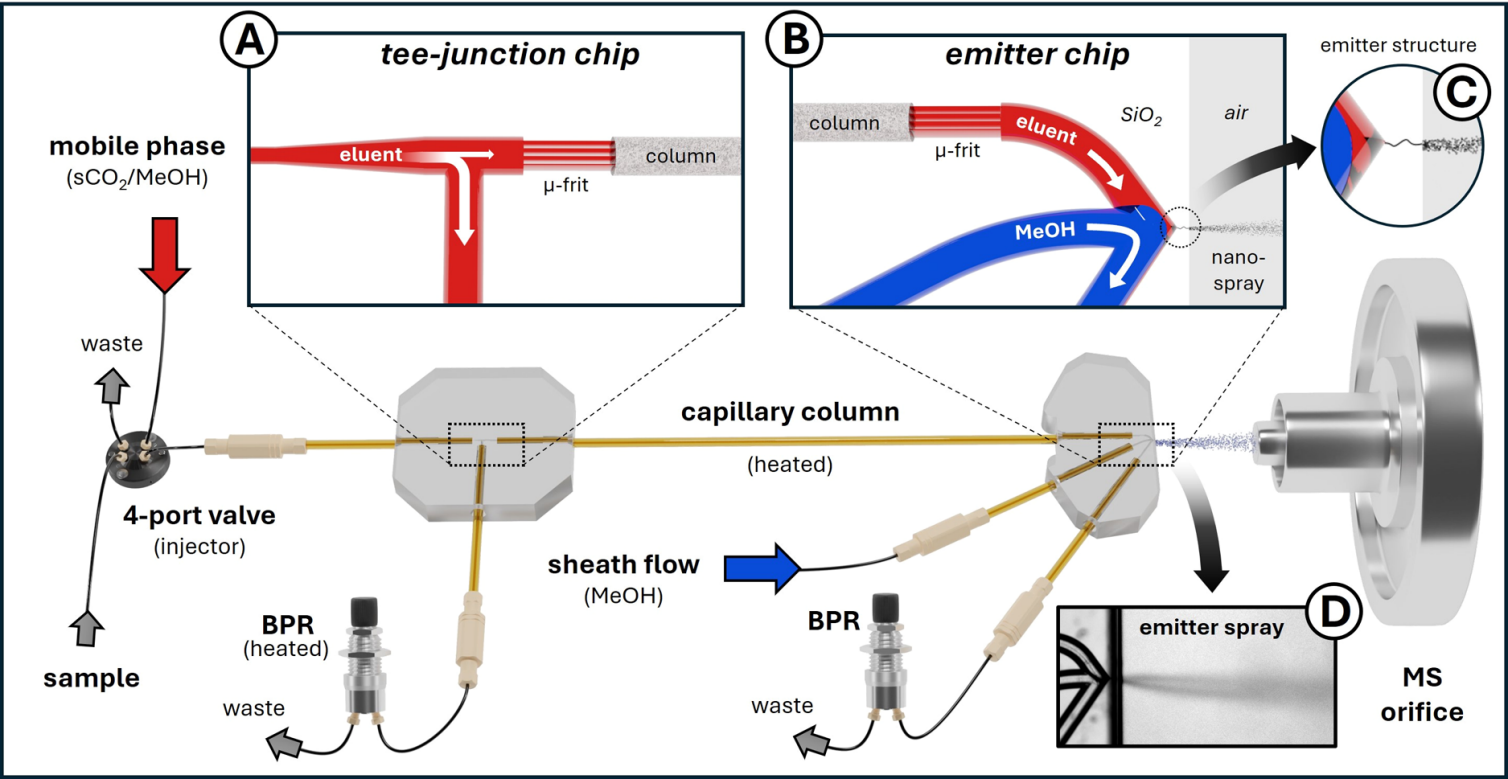
Simplified schematic representation of the SFC–MS setup (for more information see Figure S3 in the Supporting Information). From left to right: Sample injection using a 4-port valve. The internal volume of the rotor seal (4 nL) is filled with a syringe with the excess being discarded. By switching the valve, the sample is introduced into the flow of the mobile phase (highlighted in red) and transported to the tee-junction chip. (A) The flow is split toward a BPR, with only a fraction of the sample being transported onto the capillary column, where separation occurs. The analyte components elute from the column and enter the emitter chip. (B) Inside the chip, mobile phase flow and makeup flow (highlighted in blue) are joined while maintaining laminar flow. The mobile phase is compressed depending on the strength of the makeup flow and split at the emitter, with one fraction flowing toward the BPR and the other being sprayed through the emitter. (C) Decompression of CO_2_ occurs within the 8–15 μm long pinhole emitter. (D) A photograph of the spray (brightness and contrast of the image were adjusted to increase spray visibility).

The microfluidic circuitry for sample introduction, pressure control, and SFC–MS hyphenation was modified from previous work.^39^ In this setup, only 7.2% of the sample volume is loaded onto the column, as demonstrated by earlier investigations.[44] A simplified overview of the SFC–MS setup is given in Figure 1. A detailed description and overview of the components are provided in Figure S3 in the Supporting Information. Notable innovations include the reduction of dead volume in the precolumn area by using capillaries with a smaller internal diameter and a custom-made column heater (Figure S4).

### Fluorescence Measurements

Sample transport and flow distribution between the microfluidic channels adjacent to the emitter structure were evaluated using a solution of rhodamine 6G (0.1 mg/mL in MeOH). The instrumental setup and measurement conditions employed were similar to those used in standard SFC separation measurements. The emitter chip (without column and tee-junction chip) was placed on an inverted epifluorescence microscope (IX-71, Olympus, Japan) equipped with a 40× objective (LUCPLFLN 40*×*/0.6, Olympus, Japan), a LED light source (530 nm, M530L4, Thorlabs, USA), as well as an excitation filter (530/40 nm bandpass), emission filter (590 nm long pass) and dichroitic mirror (570 nm). The fluorescence signal was detected using a photomultiplier tube (H9305-03, Hamamatsu, Japan) connected to a photomultiplier controller (amplifier). Data acquisition was conducted with Clarity Chromatography Software from Data Apex (Prague, Czech Republic). A detailed overview of the setup is presented in Figure S5 in the Supporting Information.

## Results and Discussion

The platform presented in this work aims to advance the state of the art in chip-based SFC^39,44^ by integrating a pinhole emitter, a dilution-free microfluidic BPR and comb-shaped μ-frits into microfluidic chip modules. Using SLE technology, we developed monolithic fused-silica chip modules that can be easily assembled into a functional, miniaturized SFC platform, which interfaces seamlessly with atmospheric pressure ionization mass spectrometers.

**Figure 2:**
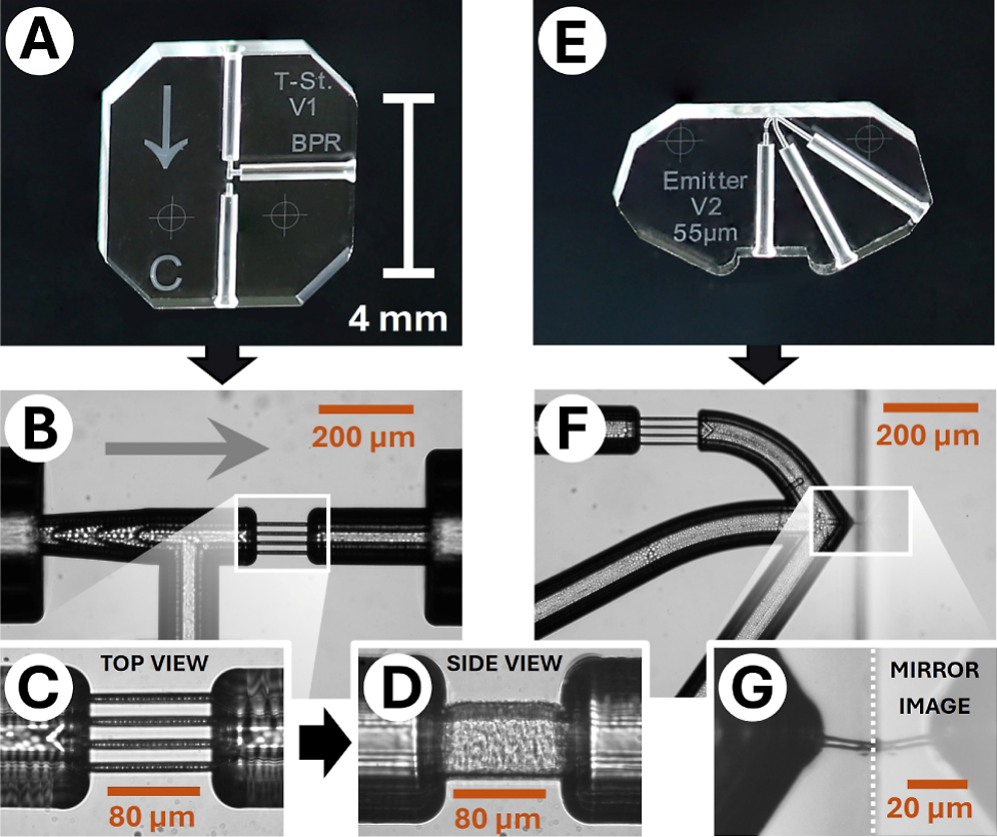
SLE fabricated chip-modules with integrated functionalities: (A) tee-junction chip with (B–D) a comb-shaped μ-frit. The arrows in (A,B) indicate the flow direction. (E) Emitter chip with (F) a μ-frit, V-shaped channel design and (G) a dielectric breakdown toward the glass edge. For better visibility, reflections and dust particles were removed from image (A,E) using GNU Image Manipulation Program.

The two chip modules, namely a flow-split and an emitter chip, were interconnected via a packed chromatographic column (ID 100 μm, variable length) and adjacent capillaries, forming the core of the novel SFC–MS platform (Figure 1). Microscopic images of the individual chips, including their microfluidic features, are depicted in Figure 2. A characteristic of both chips is the integrated retaining structure (μ-frit) that keeps the column particles within the interconnecting capillary while enabling efficient liquid transfer. Each μ-frit (Figure 2C,D) consists of four parallel 100 μm long channels with an approximate width of 7 μm and height of 74 μm. Assuming an elliptical shape, the high aspect ratios yield a cross-section equal to 20.7% of the adjacent circular channel structures (ID 100 μm). This allows for transporting low-viscosity fluids without significant pressure drop. At the same time, small particles with a diameter down to 1.6 μm are reliably retained within the μ-frits without leakage, even at elevated pressures (400 bar). Compared to common sintered^45^ or polymer frits^46^ the μ-frits are highly pressure stable, reproducible, preserve complete column functionality, are reusable and do not require error-prone manual implementation. Using a multi-step SLE process, up to 50 chip modules featuring these channel structures with single-digit μm dimensions were processed one a single wafer (Figure S1).

The tee-junction chip (Figure 2A) connected to the column inlet works as a precolumn split, as introduced earlier.^39,44^ This flow-splitting approach facilitates the use of standard macroscopic HPLC and SFC equipment, such as injection valves and solvent delivery pumps.

The emitter chip (Figure 2E) connected to the column outlet features the emitter structure and a dilution-free microfluidic BPR. Emitter development for SFC–MS coupling is crucial since decompression of scCO_2_ is often accompanied by phase separation that results in the loss of chromatographic resolution and sample precipitation. High aspect ratio capillaries are the most commonly used emitters due to their availability and durability. Nevertheless, their geometry is considered one of the worst due to the linear pressure reduction.^35^ The almost optimal geometry regarding the expansion process, namely the “pinhole” orifice, has been studied but has not seen widespread application due to practical challenges. These are the laborious fabrication process and the high susceptibility to clogging.^47^ Inspired by this, we have developed a new nanoemitter device that faces both challenges. It can be easily fabricated with a tailored monolithic SLE device by a dielectric breakdown through a thin wall^43,48^ at the edge of the fused silica substrate. The resulting nanochannel is a short (8–15 μm) and thin (approximately 1 μm) passage through the outer chip wall. It serves as a tiny edge emitter and a restrictor (Figure 2G), allowing a controlled pressure drop of more than 150 bar within a few micrometers. Since dielectric breakdowns occur at the thinnest point of the glass wall, a V-shaped channel design close to the substrate’s edge made the emitter length predictable and reproducible.

Clogging of the emitter is prevented by design: on one hand, the mobile phase reaching the emitter is already filtered by μ-frits and the column; on the other hand, only a portion of the mobile phase is transferred to the emitter, while the excess flow sweeps the emitter entry, removing solid residues. The stability of the fluidics and the gas jet at the emitter outlet was visually assessed, as illustrated in Figure S6. The examination revealed no premature phase separation inside the emitter chip, confirming that decompression of scCO_2_ predominantly occurs within the pinhole emitter structure. In addition, the spray, clearly visible under the green laser pointer illumination, showed no signs of pulsing, indicating a consistent and stable decompression process.

Another important feature of the emitter chip is the integrated dilution-free microfluidic BPR. Located in close proximity to the emitter structure, it provides pressure control while keeping postcolumn dead volume to a minimum (3 nL). This approach optimizes analyte separation by controlling the postcolumn pressure independently of the precolumn pressure set by the SFC pump. We have incorporated a makeup flow and split structure for improved pressure control. Unlike conventional methods that dilute the eluate and reduce detection sensitivity, our design directs the makeup flow to bypass the emitter, allowing the analyte to enter undiluted.

**Figure 3:**
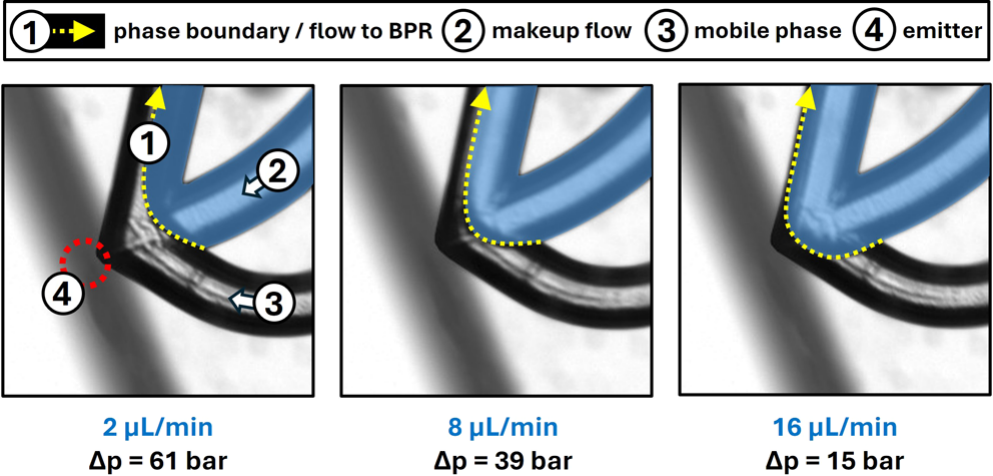
Working principle of the microfluidic BPR: as the makeup liquid (MeOH, in blue) flow rate increases, the postcolumn pressure increases accordingly, lowering the overall pressure drop along the column. At the same time, the phase boundary (yellow dotted arrow) between the mobile phase and the makeup flow moves toward the emitter structure. Meanwhile, the precolumn pressure remains unaffected (*p*_precolumn_ = 160 bar).

The working principle of this microfluidic back-pressure regulating functionality is visualized in Figure 3. The arrowhead-shaped channel structure at the emitter chip resembles a cross, with two inlets (mobile phase and makeup) and two outlets (emitter and back-pressure-stabilized split channel) positioned adjacent. Since only a limited amount of the mobile phase from the column can be transferred through the emitter, the remaining excess flows downstream to the outlet. The pressure in the postcolumn area is controlled by varying the makeup flow rate (2–16 μL/min). This minimized postcolumn volume of 3 nL allows for a rapid pressure response to changes in makeup flow.

### Evaluation of the Dilution-Free Microfluidic BPR

As the BPR integrates the makeup flow, split and emitter structure, its performance depends not only on the composition, temperature and density of the mobile phase but also on the spatial dimensions of its individual components. The behavior of this complex, multivariable system is difficult to predict and requires experimental testing within the working parameters. In this context, we have further investigated the postcolumn split ratio and the effect of column pressure drop on resolution.

For determining the split ratio, a 4 nL sample of rhodamine 6G (0.1 mg/mL in MeOH) was injected, and its fluorescence was monitored at (i) the outlet toward the BPR, (ii) the makeup inlet and (iii) the mobile phase inlet (compare Figure 3). The sample transfer through the pinhole emitter could not be registered by fluorescence microscopy due to the very small dimensions. For the measurements, the capillary column was connected directly to the SFC pump without a packed column using a mobile phase composition of 60:40 scCO_2_/MeOH and a methanol makeup flow of 10 μL/min. A detailed overview of the setup and the fluorescence measurements is illustrated in Figures S5 and S7 in the Supporting Information. Since the peak area at the outlet represents 50% (±4%) of the signal detected in the channel carrying the mobile phase, half of the sample passes through the emitter. Due to variations in the mobile phase density and flow velocity across the measuring ranges, there is a relatively high measurement uncertainty for the specified 1:1 splitting ratio.

After this set of measurements, the setup was equipped with a 4.7 cm long column of IG-3 particles to study the adjustable pressure range at actual chromatographic conditions. By gradually increasing the makeup flow, the influence on the pressure after the column and on the phase boundary between mobile phase and makeup was monitored. As model sample, a racemic mixture of *R*- and *S*-warfarin was analyzed to evaluate the impact of the pressure drop on the separation. The measurement results, shown in Figure S8, reveal that the pressure after the column increases linearly with the makeup flow rate. The median slope was found to be 3.3 bar per 1 μL/min with a minimum pressure of 99 bar at 2 μL/min and a maximum value of 145 bar at 16 μL/min, yielding an operational pressure range of 46 bar. Makeup flow rates below 2 μL/min could not be reliably sustained, while at flow rates above 16 μL/min, the makeup liquid started to enter the emitter channel as the phase boundary moved closer to the emitter structure. Adjusting the postcolumn pressure through the makeup flow, and consequently the pressure dropalong the column, directly influenced the chromatographic resolution, thereby demonstrating the practical effectiveness of the microfluidic BPR functionality (Figure S9). Increasing the makeup flow rate from 2 μL/min up to 16 μL/min improved *R*_S_ values from 1.16 to 2.06. Meanwhile, the reduced pressure drop increased analysis time and diminished signal intensity. The slower flow of the mobile phase also increased residence time, amplifying longitudinal diffusion and causing fwhm values to rise by 63–67%. It should be noted that the change in postcolumn pressure can also influence the split ratio in the precolumn area as described in more detail in the Supporting Information.

### Optimizing Spray Voltage for MS Sensitivity

The influence of the spray voltage on the MS signal response was also investigated as part of method development. It should be noted here that the glass emitter is not electrically contacted. In general, the ionization process in SFC–MS couplings under direct expansion of the mobile phase is scarcely researched^49^ and remains unclear. It was also not the focus of this study. Instead, the aim was to investigate how much the MS inlet voltage influences the sensitivity, e.g., through improved ion transmission into the mass spectrometer. To this end, a racemic mixture of warfarin was separated using a packed IG-3 column (ID 100 μm, 4.7 cm) in a set of experiments at varying MS inlet voltages between −2.5 and −6 kV. Other separation parameters were kept equal. The results are presented in Figure S10 in the Supporting Information. It was observed that starting from −2.5 kV, the signal intensity and signal area strongly increased to a maximum of −4.5 kV. Beyond this point, the signal parameters declined slowly. Based on these results, an MS inlet voltage of −4.5 kV was employed for further experiments.

### Chiral Separations of Pharmaceuticals and Synthetic Substances

**Figure 4:**
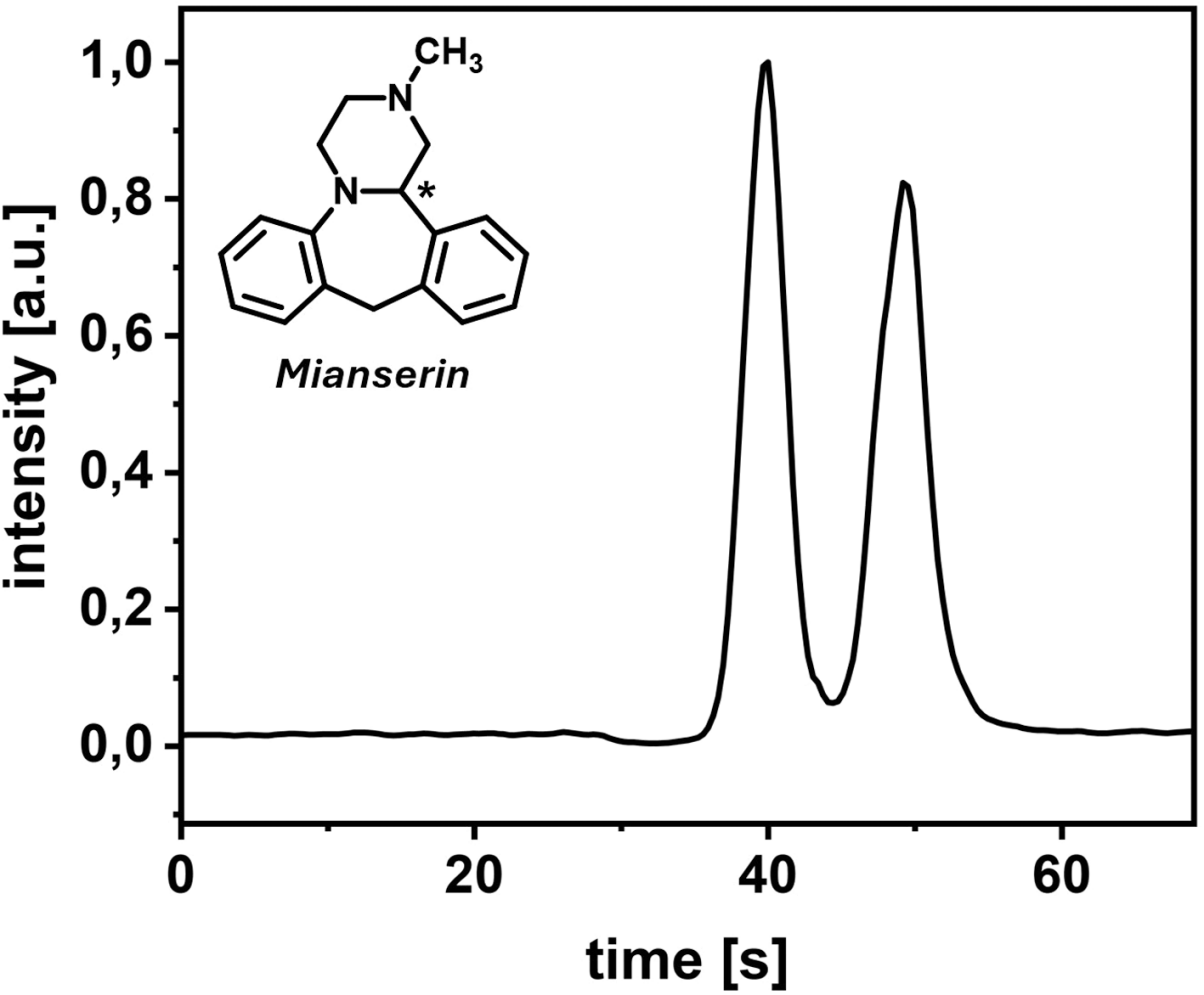
SFC–MS chromatogram (EIC) of mianserin. Sample: 4 nL of 1 mM mianserin dissolved in MeOH; mobile phase: 80:20 v/v CO_2_/MeOH (0.1% FA); column: 5.2 cm, IG-3, *T* = 40 °C, *p*_precolumn_ = 112 bar, *p*_postcolumn_ = 95 bar; MeOH makeup flow: 4 μL/min; MS: 2.9 Hz, positive mode, −4.5 kV.

**Figure 5:**
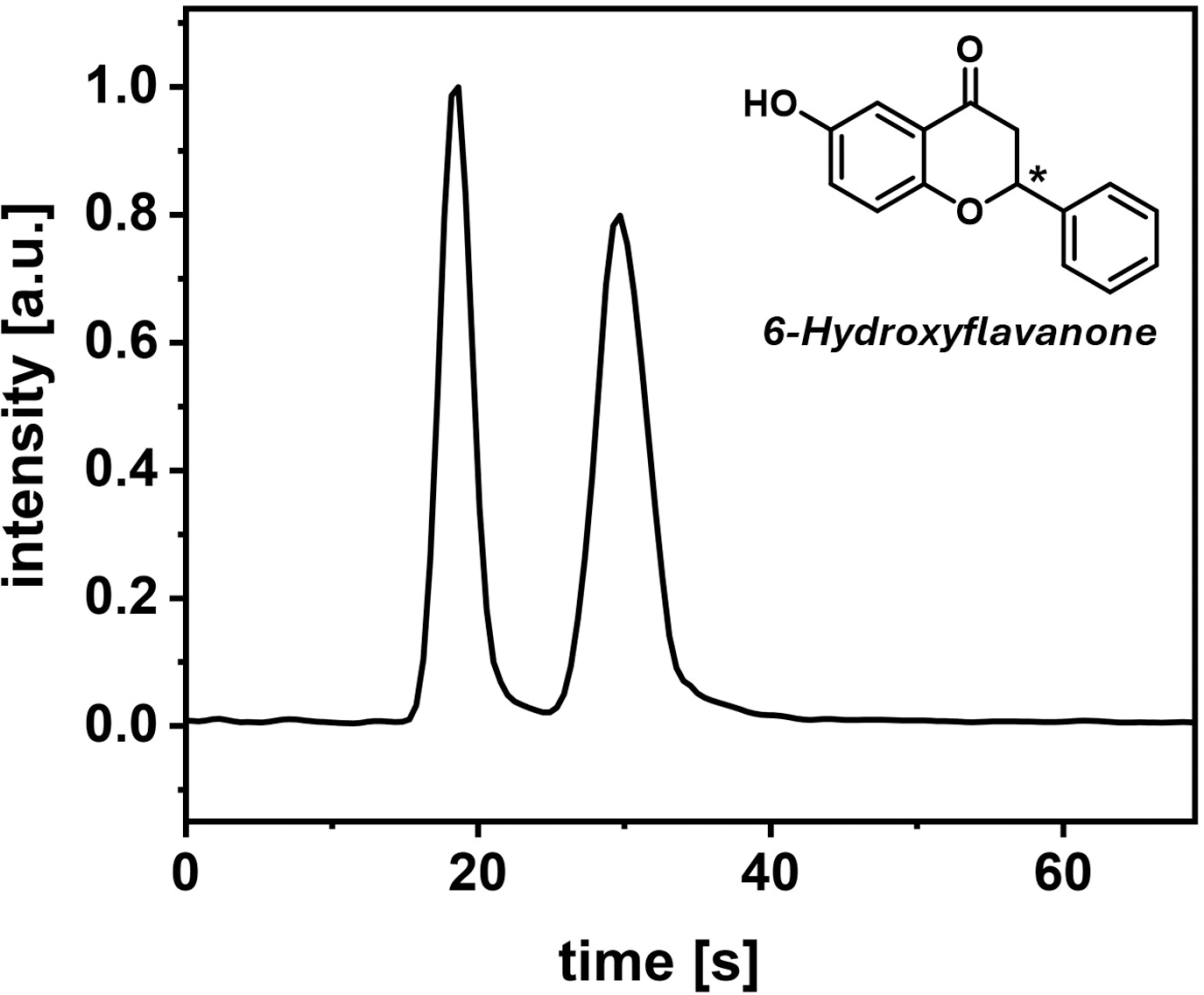
SFC–MS chromatogram (EIC) of 6-hydroxyflavanone. Sample: 4 nL of 1.0 mM 6-hydroxyflavanone dissolved in MeOH; mobile phase: 75:25 v/v CO_2_/(MeOH/*i*PrOH/NH_3_ with ratio 50:50:0.1); column: 4.0 cm, IA-3, *T* = 28 °C, *p*_precolumn_ = 193 bar, *p*_postcolumn_ = 149 bar; MeOH makeup flow: 5 μL/min; MS: 2.1 Hz, negative mode, 3.0 kV.

During the evaluation of the SFC–MS platform, the separation of *R*- and *S*-warfarin was successfully conducted, proving the usability of the setup for fast chiral analysis. In an extended chiral SFC–MS analysis study focusing on separation speed and reproducibility, the chiral drugs mianserin and 6-hydroxyflavanone were analyzed. The separation of *R*- and *S*-mianserin was achieved via SFC–MS in less than a minute using an IG-3 column (5.2 cm, ID 100 μm). The corresponding chromatogram is shown in Figure 4. Chromatographic performance parameters are listed in Table 1. The peak analysis of five consecutive measurements revealed a median *R*_s_-value of 1.70 (±0.08), indicating complete enantiomer separation. Low RSD values (<1%) of the according retention times prove high reproducibility despite fast analysis. The deviation of integrated peak area (RSD 7.4% and 7.5%) is inherently higher but sufficiently low to allow for substance quantification. Unlike mianserin, no signal was detected for 6-hydroxyflavanone in positive mode, leading to a switch to negative mode. The MS inlet voltage was set to 3.0 kV, and 0.1% ammonia was added to support deprotonation of the analyte. In this way, baseline separation of the enantiomers (*R*_s_ = 2.1) was achieved in less than 40 s. Chromatogram and performance parameters are presented in Figure 5 and Table 2.

**Table 1:** Chromatographic Performance Parameters of Mianserin Separation

peak no.	retention time [s]^a^	fwhm [s]^a^	*R*_s_^a^
1	42.0 (0.8%)	3.4 (7.0%)	
2	52.6 (0.8%)	3.9 (5.7%)	1.70 (4.7%)

a Results presented as mean (RSD in %) of five measurements.

**Table 2:** Chromatographic Performance Parameters of 6-Hydroxyflavanone Separation

peak no.	retention time [s]^a^	fwhm [s]^a^	*R*_s_^a^
1	19.1 (2.7%)	2.6 (9.4%)	
2	31.3 (3.5%)	4.4 (7.4%)	2.10 (6.8%)

a Results presented as mean (RSD in %) of three measurements.

### Application: Detection and Quantification of Tocopherols in Potato Chips

**Figure 6:**
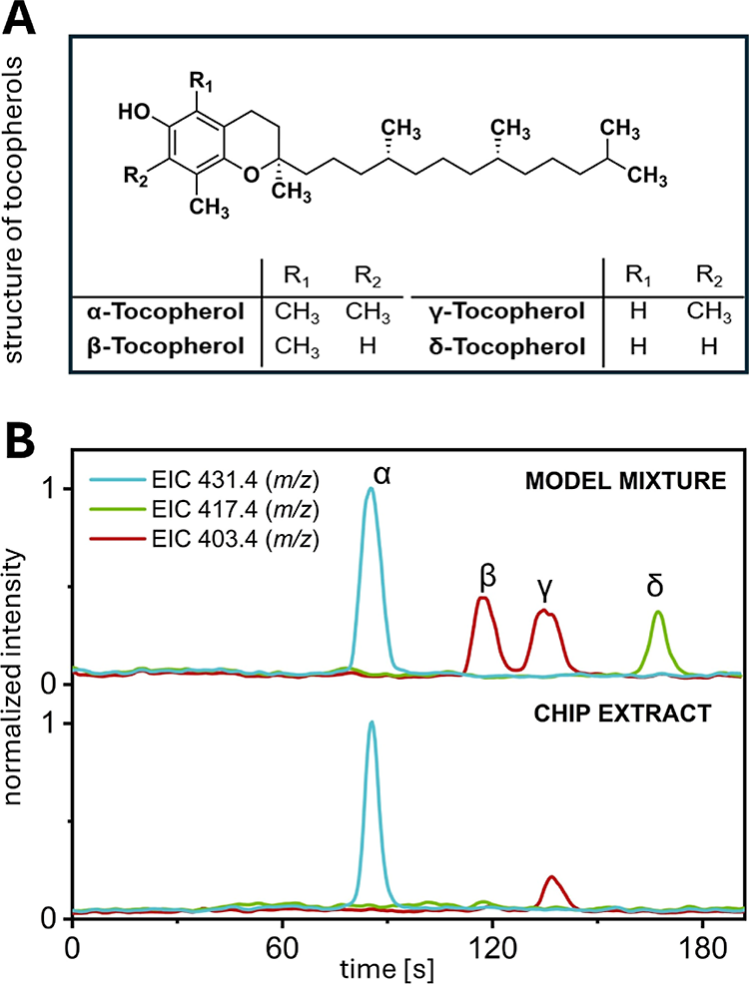
Analysis of tocopherols in potato chip extract. (A) Structural formulas of different types of tocopherols. (B) SFC–MS chromatogram (EIC) of the tocopherol model mixture (top) and potato chip extract (bottom). Sample: 4 nL of 1 mM α-, β-, γ- and δ-tocopherol dissolved in MeOH (top) or chip extract (bottom); mobile phase: 98:2 v/v CO_2_/MeOH (0.1% FA); column: 8.7 cm, silica (*d*_p_ = 2.2 μm), *T* = 55 °C, *p*_precolumn_ = 173 bar, *p*_postcolumn_ = 106 bar; makeup flow: 14 μL/min; MS: 2.4 Hz, positive mode, −4.5 kV inlet, 250 V fragmentor voltage. For better visibility, signals were processed with a 15 point lowess filter.

Following the successful use of the SFC–MS platform for high-speed chiral analysis, we also used the technology to analyze a food sample regarding its tocopherol content. Tocopherols occur in four forms: α-, β-, γ- and δ-tocopherol. They differ only in the number and their position of methyl groups adjacent to the hydroxy group in the 6-hydroxychromane backbone (Figure 6A).

To assess the tocopherol content in potato chips, a sample solution was prepared using the solvent extraction and saponification methods described in detail in the experimental section. To identify the different tocopherols by their elution times and distinguish between the diastereomers β- and γ-tocopherol, a model mixture containing all tocopherol forms was analyzed first. Isocratic separation of these components was achieved within 3 min using bare silica as the stationary phase (Figure 6B). A mixture of scCO_2_ and MeOH (0.1% FA) in a ratio of 98:2 was used as mobile phase. For the closely spaced β- and γ-tocopherol signals, a *R*_S_ value of 1.45 was obtained, which was adequate for their individual identification. In the next step, the potato chip extract was analyzed under the same conditions, revealing the presence of α-tocopherol and γ-tocopherol, with γ-tocopherol detected in significantly lower amounts. Due to γ-tocopherol’s weak signal intensity, which was near the limit of detection (LOD) at 10 μM, only α-tocopherol, with a lower LOD of 2.5 μM, could be quantified. The LOD values for β- and δ-tocopherol were found to be 10 and 30 μM, respectively. To estimate the amount of α-tocopherol in the chip extract, known solutions of α-tocopherol with concentrations ranging from 10 to 200 μM were analyzed, using the parameters stated in Figure S11. Linear regression of the peak area produced an *R*^2^ value of 0.99. Based on five consecutive measurements of the extract under identical conditions, the amount of α-tocopherol in the sample was calculated to be approximately 36 μM ± 6 μM, equivalent to around 8 mg per 100 g of potato chips. Since matrix effects were not taken into account, this value is considered a rough estimation. However, the result aligns well with the current maximum amount proposed by the German Federal Institute for Risk Assessment of 7 mg per 100 g for solid foods.^50^

## Conclusions

In this work, we present the development and evaluation of a novel SFC–MS platform using SLE fabrication techniques with advanced integrated functionalities for simplified assembly and interfacing. Key features include μ-frit retaining structures, a pinhole emitter and a dilution-free microfluidic BPR. The system is characterized by a high degree of flexibility in pressure regulation, facilitating separation optimization without compromising sample integrity. Fast chiral separations in less than 60 s in both positive and negative MS modes highlight the platform’s broad applicability in high-throughput methods. In addition, the detection, identification and quantification of tocopherols in a sample of potato chips demonstrated low detection limits at fast separations. The nL volume back-pressure regulation unit is effective in directed optimization of chromatographic performance and avoids analyte dilution. This novel technology thus offers an excellent and versatile approach that overcomes the limitations of established SFC–MS systems.

## Supporting Information Available

Detailed information about the SLE manufacturing process, the chipSFC–MS assembly and setup (incl. fluorescence measurements). Images of the spray, supplementary results regarding postcolumn split ratio, makeup-controlled pressure control, spray voltage and tocopherol quantification (PDF)
