# Supplementary material for: Novel Pinhole Emitter Chip for Micro Supercritical Fluid Chromatography–Mass Spectrometry with Integrated Dilution-Free Fluidic Back-Pressure Regulation
Source: Anal Chem. 2024 Dec 2;96(50):20107–14. doi: 10.1021/acs.analchem.4c05171 (PMC11656411; doi:10.1021/acs.analchem.4c05171)
Supplement: Supplementary file 1 — ac4c05171_si_001.pdf [file ac4c05171_si_001.pdf]

SUPPORTING INFORMATION

Novel Pinhole Emitter Chip for Micro Supercritical Fluid Chromatography-Mass Spectrometry with Integrated Dilution-Free Fluidic Back-Pressure Regulation

Julius Schwieger, Chris Weise and Detlev Belder*

Institute of Analytical Chemistry, Leipzig University, Linnéstraße 3, 04103 Leipzig, Germany

Corresponding author:

*E-mail Detlev Belder: belder@uni-leipzig.de

Table of content

Figure S1	–	Overview of the chip-manufacturing process	page – S2
Figure S2	–	Assembly of the nanoscale SFC-system	page – S3
Figure S3	–	Schematic overview of the instrumental SFC setup	page – S4
Figure S4	–	Overview of the chip positioning and the heating system at the MS inlet	page – S5
Figure S5	–	Instrumental setup for fluorescence measurements	page – S6
Figure S6	–	Images of the SFC-MS interface and emitter spray	page – S7
Figure S7	–	Investigation of the split ratio on the emitter chip using fluorescence measurements	page – S8
Figure S8	–	Evaluation of the adjustable pressure range of the microfluidic BPR	page – S9
Figure S9	–	Influence of the microfluidic BPR to the separation resolution	page – S10
Figure S10	–	Evaluation of peak area and signal intensity depending on the MS inlet voltage	page – S11
Figure S11	–	Calibration curve for the quantification of α-tocopherol	page – S12

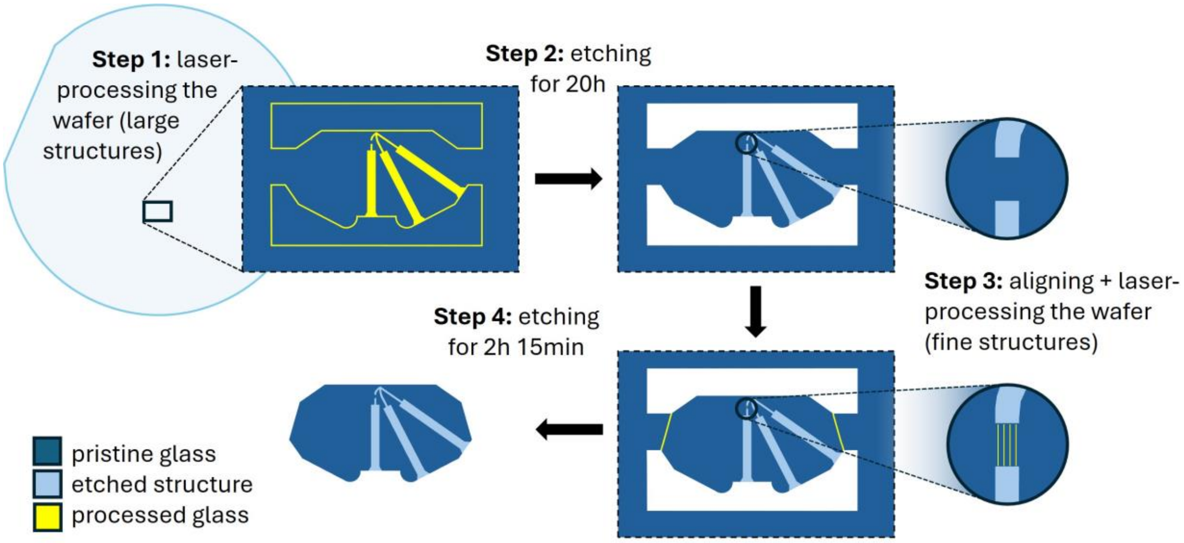

Figure S1 - Overview of the chip-manufacturing process – The chip manufacturing consists of four individual steps; both structuring and etching are done twice. For structuring a 400 fs Yb:YAG-laser (λ = 1030 nm) was focused through a 20x objective (LHM-20X-1064, NA = 0.40, Thorlabs, Newton, NJ, USA) onto a polished fused silica wafer (1mm, 4 inch diameter, UV grade, T24008, purchased from Siegert Wafer, Aachen, Germany). The laser energy (160, 230, 250, 460 nJ) and the layer-distance in X-, Y- and Z-direction (3, 5 or 7 µm) were chosen according to each structural element. The repetition rate was set to 1 MHz, the feed rate was 15.83 mm/s, and the polarization was perpendicular to the feed rate vector. For etching, the substrate was submerged in 1 liter of hot (84 °C) 8 M KOH solution. Then, 5 mL of methanolic dioctyl sulfosuccinate sodium solution was added, resulting in a total surfactant concentration of 0.02 wt%. The manufacturing procedure (structuring and etching) is identical for the emitter chip (as shown above) and the tee-junction chip. Therefore, both can be fabricated simultaneously in higher quantities within a single wafer (approx. 50 pieces). **Step 1:** microfluidic channels, inlet structures for the microfluidic connection via fused silica capillaries and parts of the outline are structured inside the glass. Additional aligning structures (not shown in the figure) are added at the right and left sides of the wafer. **Step 2:** The wafer is washed with water, then deionized water and lowered into a box containing 1 L of degassed aqueous 8 M KOH (sonicated for 20 min) and 5 mL of dioctyl sulfosuccinate solution in MeOH at 84 °C. The box is placed inside a heated ultrasonic bath, ensuring a constant temperature during etching. The wafer remains in the etching solution for 20 hours. A timer ensures sonication for 120 seconds each 13 minutes. **Step 3:** The wafer is removed from the etching solution, sonicated in deionized water for 30 minutes and dried on a hotplate at 90 °C. The wafer is then again mounted in the SLE-device and aligned using the corresponding structures. The remaining outline of the chip, as well as the retainment structure (µ-frit), are processed. **Step 4:** The wafer is etched for 135 minutes in a fresh etching solution. During the first hour, it is sonicated constantly to guarantee the reentry of the etching solution inside the microfluidic structures. Afterwards, it is sonicated periodically (2 min on, 13 min off). The etching solution is discarded, and the box with the wafer and the glass chips is refilled with deionized water twice. Some chips may still be fixed in the wafer, but they can be seperated by applying gentle pressure. The chips are then taken out and the liquid inside the microfluidic channels is removed using an air pistol. The chips are then put inside a casting solvent (6:2:2 acetonitrile, ethanol, aqueous 5mM phosphate buffer) for 15 minutes. Subsequently, the liquid inside the microfluidic channels is removed again using an air pistol. Finally, the parts are fully dried on a hotplate at 90 °C.

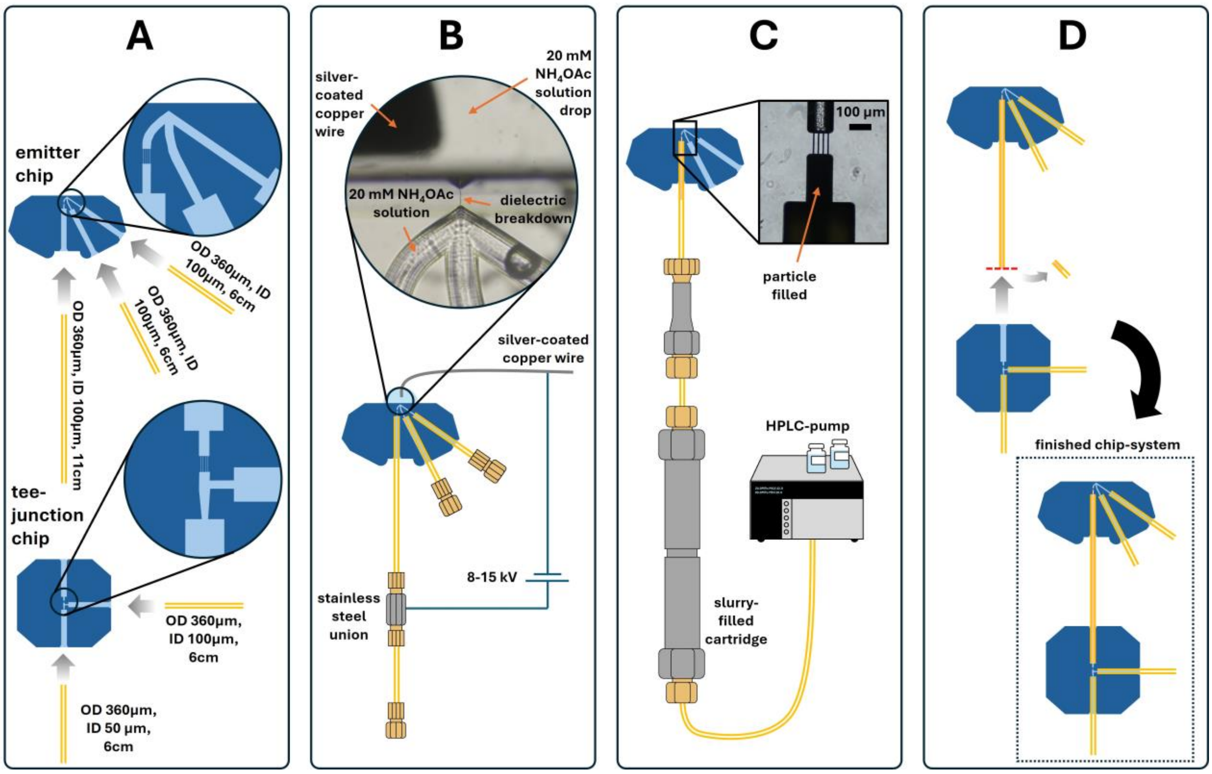

Figure S2 – Assembly of the nanoscale SFC-system – (**A**) The SLE fabricated chips were equipped with capillaries of different lengths and inner diameter (360 µm OD, Postnova Analytics, Landsberg, Germany). The capillary ends were polished, cleaned and glued into the inlet structures. (**B**) To generate the dielectric breakdown, the microfluidic channels of the emitter were filled with an aqueous solution of 20 mM NH_4Ac. The column capillary was equipped with a stainless-steel union (100 µm ID, VICI AG International, Schenkon, Switzerland) to apply a potential to the liquid via a high voltage power supply (HCN 35-35000 from FUG Elektronik, Schechen, Germany). A grounded silver-coated copper wire was aligned towards the channel joint, and a drop of the NH_4Ac solution was placed between the wire and the chips' edge. The dielectric breakdown occurred upon a voltage of 8-15 kV, accompanied by a vigorous formation of gas bubbles inside the channel joint. (**C**) The emitter chip was connected to a pump with the slurry-filled cartridge in between. At constant pressure mode (35 MPa), the column was packed. (**D**) The particle-filled capillary was cut to the desired length and subsequently glued into the tee-junction chip.

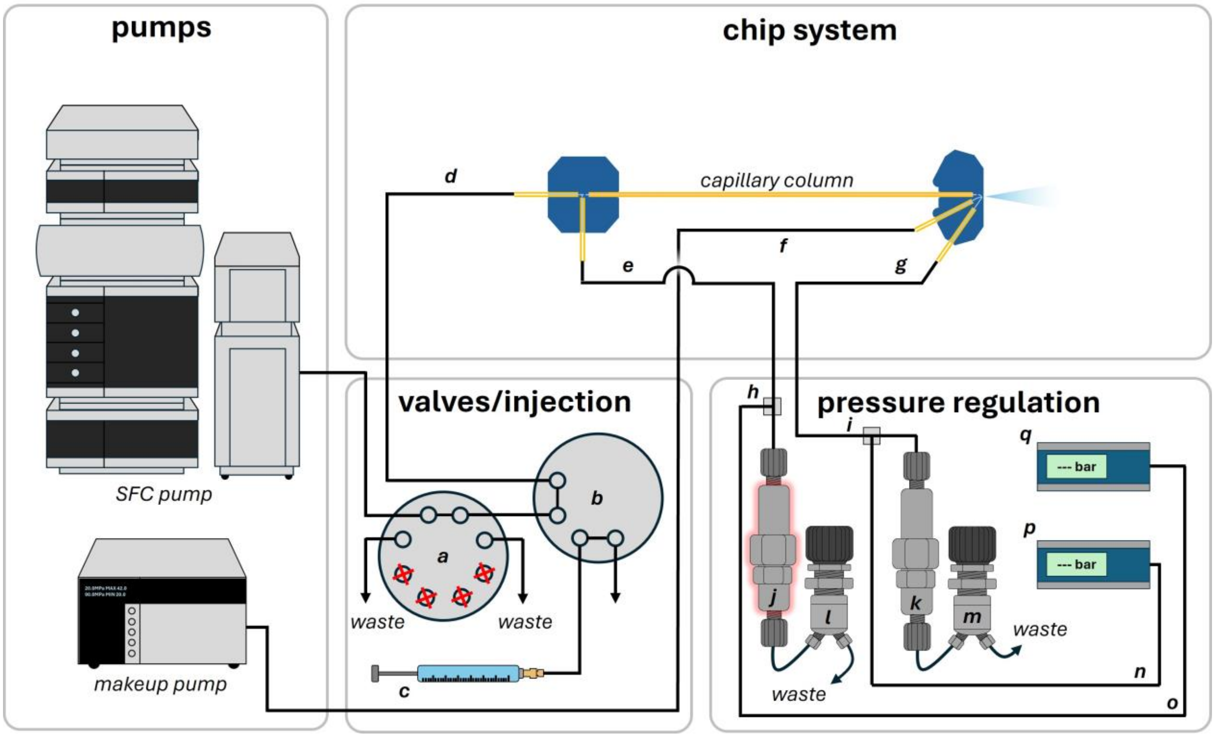

Figure S3 – Schematic overview of the instrumental SFC setup – (**a**) 8-port valve (Cheminert, VICI, Schenkon, Switzerland), (**b**) nano volume injection valve (4nL injection volume, nanoinjection valve C74MPKH, 4-port, 2-position, VICI AG International, Schenkon, Switzerland), (**c**) glass syringe (100 µL Hamilton, Switzerland) equipped with a PEEK needle port (VICI, Schenkon, Switzerland), (**d**) PEEK, OD 360 µm, ID 50 µm, 15 cm, (**e**) PEEK, OD 360 nm, ID 100 µm, 31 cm, (**f**) PEEK, OD 360 µm, ID 100 µm, 40 cm, (**g**) PEEK, OD 360 µm, ID 100 µm, 21 cm, (**h**, **i**) quick mount Tee, 100 µm, PEEK (VICI, Schenkon, Switzerland), (**j**) static BPR, 1000 psi, 50 °C using a heater cartridge (Caloratherm, Selerity Technologies, Salt Lake City, UT-USA) (**k**) static BPR, 1000 psi, (**l, m**) dynamic BPR "JR-BPR3" 1300-4200 psi (VICI, Schenkon, Switzerland) (**n**) PEEK, OD 360 µm, ID 100 µm, 37 cm, (**o**) PEEK, OD 360 µm, ID 100 µm, 26 cm, (**p**, **q**) pressure sensor (Duratec, Hockenheim, Germany).

Description: The chip-based SFC-MS platform was connected to the SFC pump for the mobile phase supply via two valves. The first valve (**a**) enabled to switch the mobile phase feed on and off. This allowed the pump to run while the chip system was being set up or exchanged, as the mobile phase can be channeled to the waste via a restrictor element. The second valve (**b**) was used for sample injection. It was equipped with an internal rotor seal (4 nL volume) that was filled manually using a glass syringe (**c**). Switching the valve introduced the sample plug into the mobile phase flow and transported it towards the column head on the tee-junction chip. To reduce precolumn dead volume, the ID of the connecting capillary (**d**) was reduced to 50 µm. A tapered channel structure inside the tee-junction chip provided a seamless transition to the larger channel structures while maintaining sample plug integrity. Two BPR assemblies connected to the glass chips via PEEK capillaries (**e, g**) effectively achieved pressure stabilization within the chip system. Each assembly consisted of a sequential connection of a static (**j**, **k**) and dynamic (**l, m**) BPR. The electronic acquisition of pressures in the pre- and postcolumn areas was achieved using two sensors (**p**, **q**) connected to the pressure stabilization assembly via T-pieces (**h**, **i**) and PEEK capillaries (**n**, **o**). While the pressure in the precolumn area was set directly at the SFC pump, pressure control in the postcolumn area was achieved by adjusting the flow rate of the makeup-liquid (MeOH, delivered by the HPLC pump LC-20AD from Shimadzu, Kyoto, Japan) that is flowing towards the emitter chip via a PEEK capillary (**f**). The fluidics close to the interface were monitored visually by observing the phase boundary between the mobile phase and makeup liquid using the AxioCam 503 camera (Zeiss, Oberkochen, Germany) equipped with the 10x lens Z16 APO from Leica (Wetzlar, Germany). Valve switching, triggering MS acquisition and pressure readouts were monitored using Clarity Chromatography Software from Data Apex (Prague, Czech Republic).

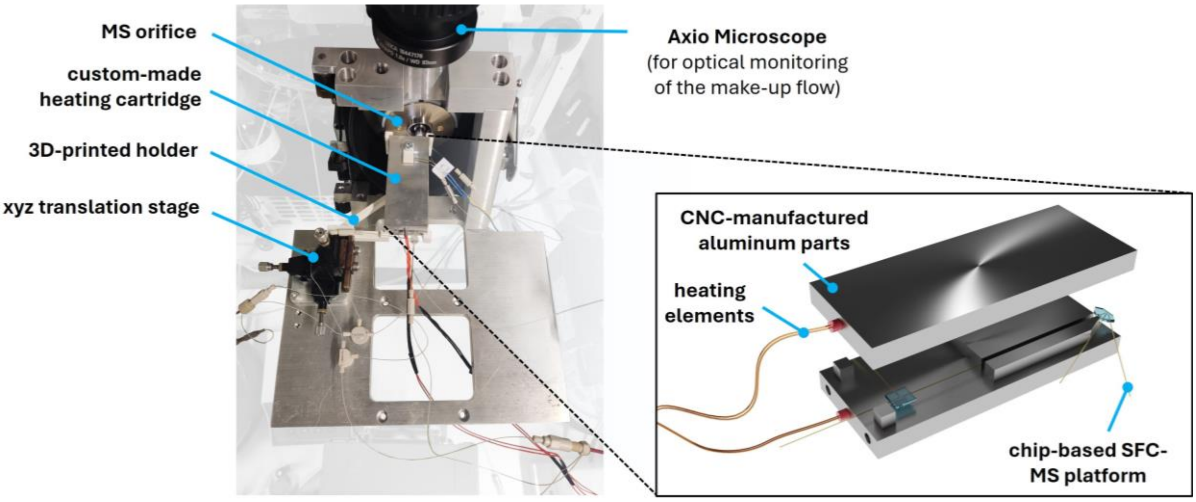

Figure S4 – Overview of the chip positioning and the heating system at the MS inlet – For column heating and temperature control, the microscale SFC system was enclosed into a custom-made aluminum housing equipped with two cylindrical heating cartridges (T+H 1116 HLP, 24V, 20W) from Türk+Hillinger (Tuttlingen, Germany). The emitter chip was kept visible. Temperature control (25-60°C) was achieved using a PID (ESM-4420) from EMKO (Bursa, Turkey) equipped with a power supply (BASETech BT-305, Conrad Electronics, Hirschau, Germany) and a miniature temperature sensor (S651PDY24A. Minco, Minneapolis, MN-USA).

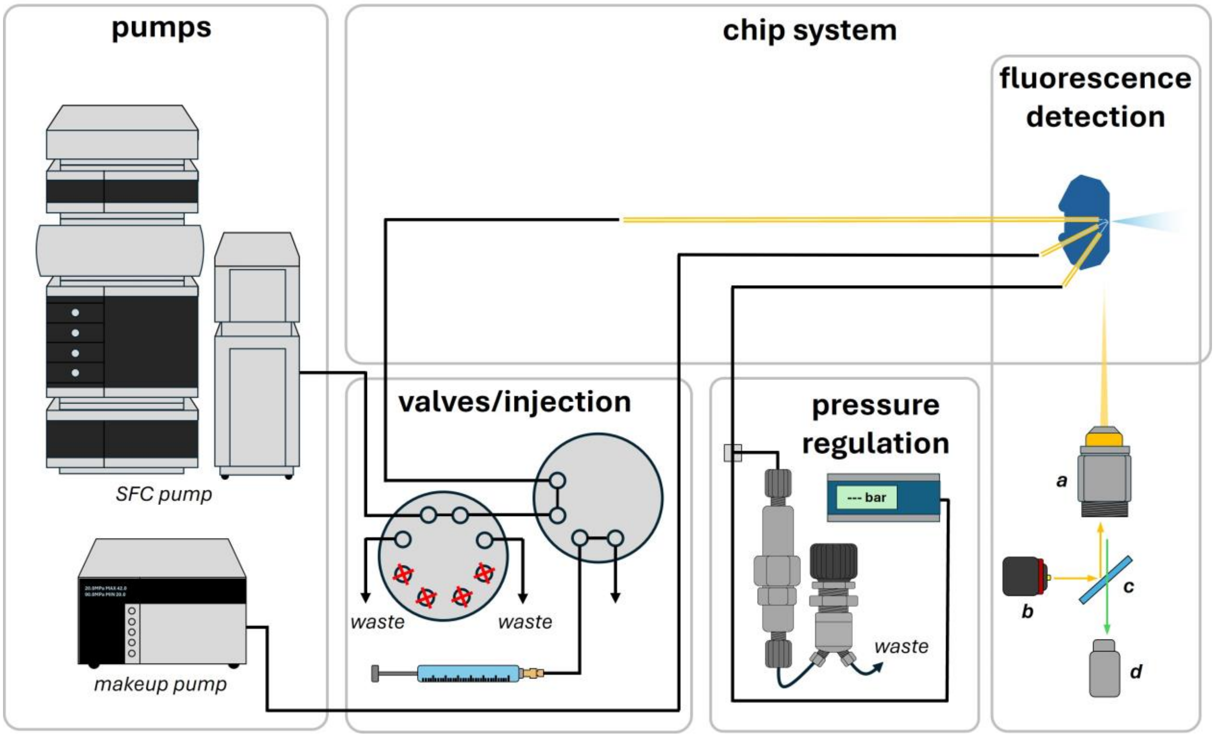

Figure S5 – Instrumental setup for fluorescence measurements – For fluorescence measurements, the SFC setup shown in Figure S3 was adapted and complemented with an epifluorescence microscope (IX-71, Olympus, Japan). Since only the flow behavior of the injected sample in the vicinity of the emitter structure was to be investigated, no column and therefore no T-junction chip was required. Hence, the flow split system in the precolumn area was omitted. The additional instrumentation employed for the fluorescence detection is listed in the following: (**a**) 40x objective (LUCPLFLN 40x/0.6, Olympus, Japan) (**b**) LED light source (530 nm, M530L4, Thorlabs, USA), (**c**) excitation filter (530/40 nm bandpass), emission filter (590 nm long pass) and dichroitic mirror (570 nm), (**d**) photomultiplier tube (H7711-03, Hamamatsu, Japan) connected to a photomultiplier controller (amplifier).

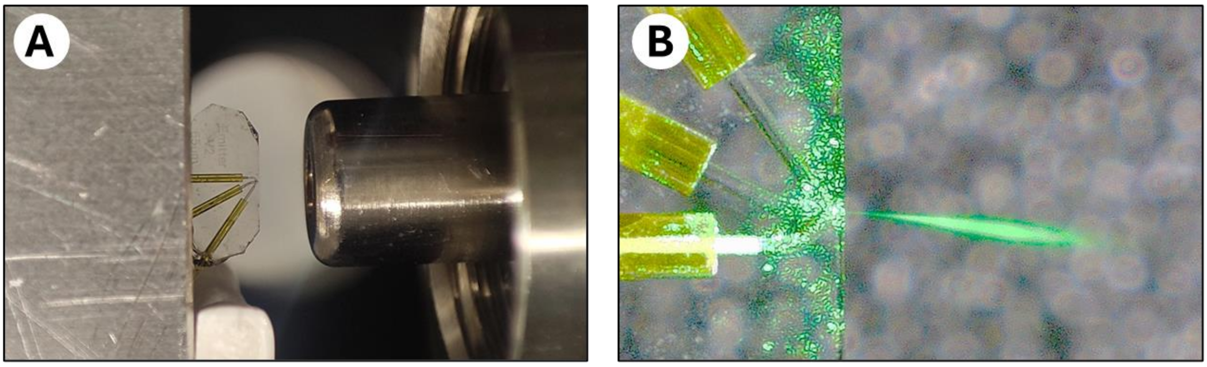

Figure S6 – Images of the SFC-MS interface and emitter spray – (A) positioning of the emitter at the MS orifice. The emitter chip protrudes a few millimeters from the aluminum housing of the electric column heater. The spray is not visible to the naked eye. (B) The edge of the emitter chip is illuminated by a green laser pointer, revealing a stable spray formed by the decompression of the CO_2-based mobile phase.

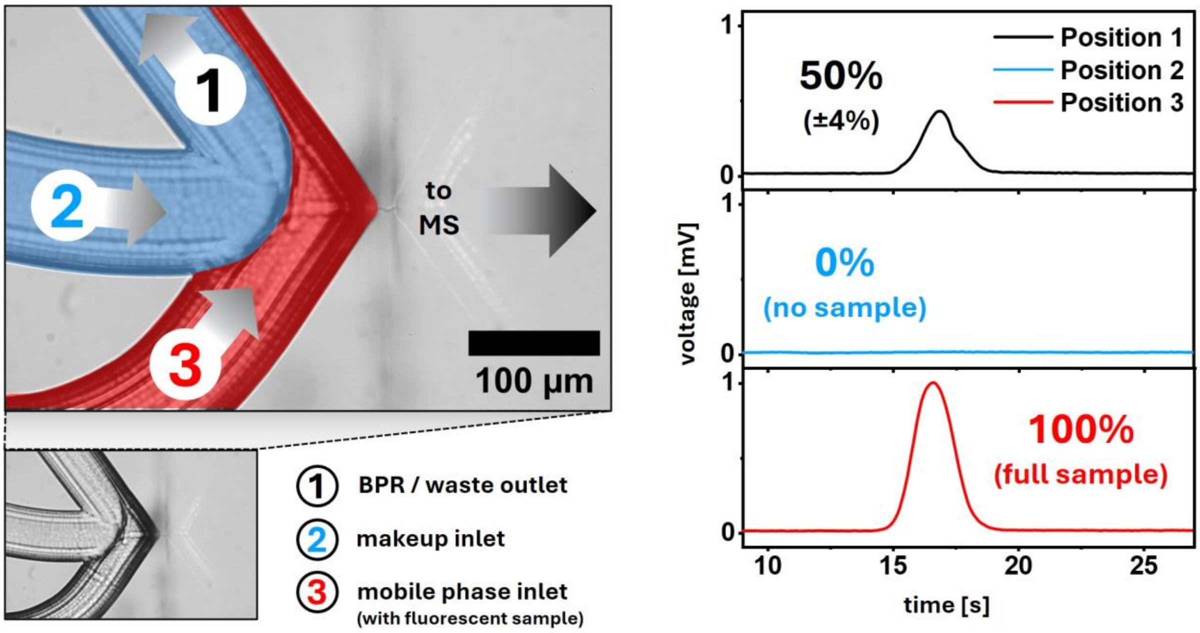

Figure S7 – Investigation of the split ratio on the emitter chip using fluorescence measurements – For the evaluation of the split ratio, the fluorescence intensity of an injected sample plug (4 µL rhodamine 6G solution, 0.1 mg/ml in MeOH) was measured three times (n=3) at the three areas of the channel joint inside the emitter chip: (1) outlet towards BPR/waste, (2) makeup/MeOH inlet (3) mobile phase inlet. The mobile phase composition was 60:40 v/v CO_2:MeOH (0.1% FA) to provide sufficient solubility of the sample. The pressure at the SFC pump (mobile phase inflow) was set to 100 bar, the pressure measured at the outflow was 99 bar. No column was used. The MeOH makeup flow was set to 10 µL/min. The peak area at the outlet equaled 50% (±4%) of the peak area measured at the mobile phase inlet.

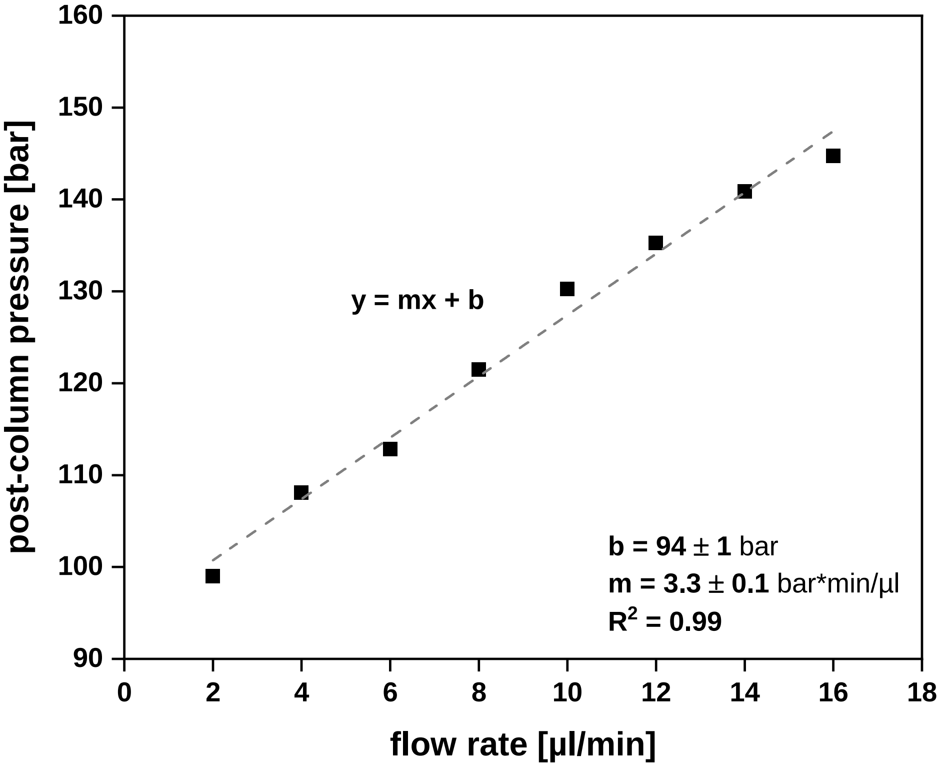

Figure S8 – Evaluation of the adjustable pressure range of the microfluidic BPR – The MeOH makeup flow towards the emitter chip was changed, while measuring the pressure at the outflow towards the BPR. Following each adjustment to the makeup flow rate, the system was allowed a three-minute equilibration period to ensure stability. The recorded pressure was subsequently determined by averaging the pressure readings over the next two minutes. The following setup parameters were employed: mobile phase: 70:30 v/v CO_2:MeOH (0.1% FA); column: 4.7 cm, IG-3, t = 40 °C, precolumn p = 160 bar; MeOH makeup flow: 2-16 µL/min; MS: positive mode, −4.5 kV.

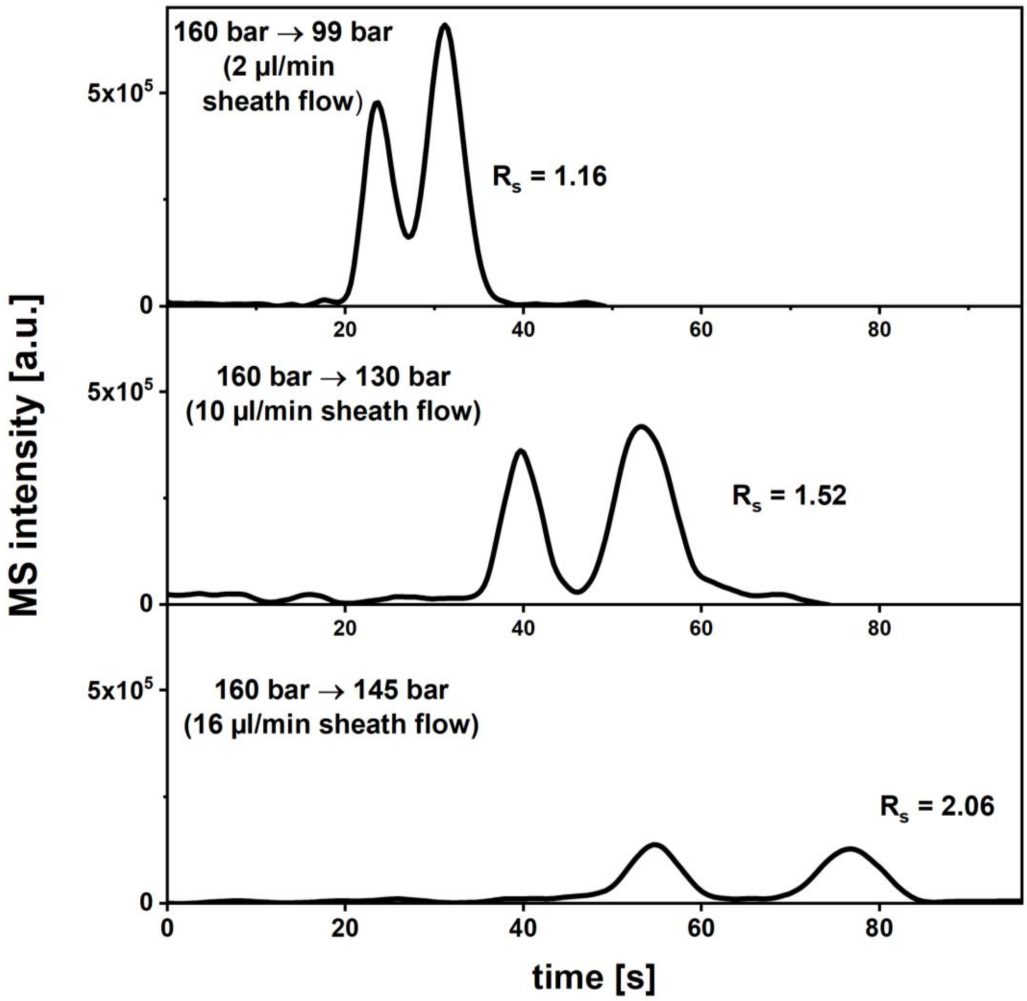

Figure S9 – Influence of the microfluidic BPR on the separation resolution – The presented chromatograms (EIC) refer to the measurements in Figure S8. Chiral separation of a racemic Warfarin mixture (1mM dissolved in MeOH) was performed under constant precolumn pressure (160 bar), while postcolumn pressures were varied. The postcolumn pressure adjustments were achieved exclusively by modifying the MeOH makeup flow rate. Measuring parameters are listed below Figure S8. Chromatograms were processed with a 13 point lowess filter. The reduction in signal intensity as pressure difference and flow rate along the column decreases can be attributed to two factors: First, according to the Van Deemter equation, a lower flow rate is associated with increased longitudinal diffusion as residence time increases (B term). Therefore, the signals broaden and lose intensity. Secondly, changes in the flow rate along the column also affect the precolumn split ratio. Since the precolumn pressure is constant, the flow split to the BPR is identical in all measurements. However, since the fraction of the sample plug that reaches the column head is determined by the ratio of the flow rates to the BPR and to the column, the effective amount of sample is reduced as the flow rate along the column decreases. The reduction in sample volume might then lead to a reduction in MS signal response.

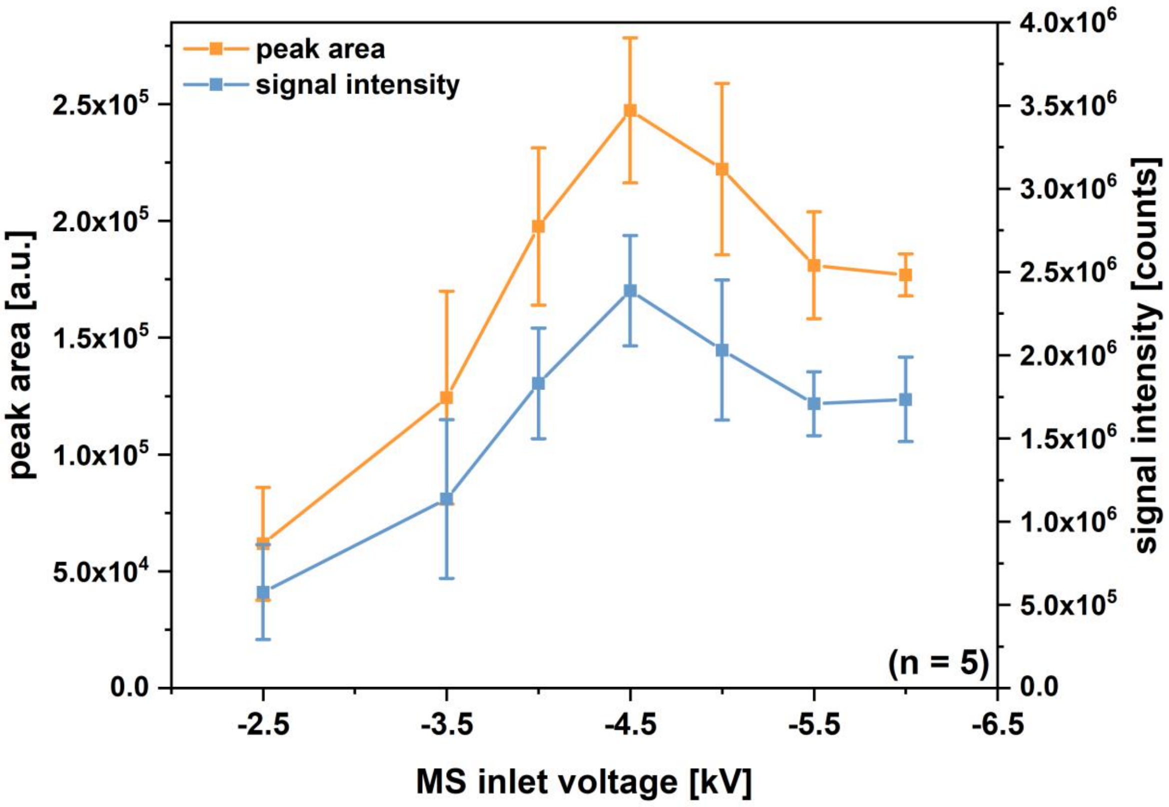

Figure S10 – Evaluation of peak area and signal intensity depending on the MS inlet voltage – For the investigation of the relationship between MS inlet voltage and peak area or peak intensity, a chiral separation of a racemic Warfarin mixture (1mM dissolved in MeOH) was performed (Rs ≥ 1.5). The MS inlet voltage was varied while all other parameters were kept constant: mobile phase: 70:30 v/v CO_2:MeOH (0.1% FA); column: 4.7 cm, IG-3, T = 40 °C, $p_{precolumn}$ = 166 bar, $p_{postcolumn}$ = 127 bar; MeOH makeup flow: 10 µL/min; MS: positive mode, −4.5 kV. The parameters were calculated based on the later eluting enantiomer by fitting Gaussian curves.

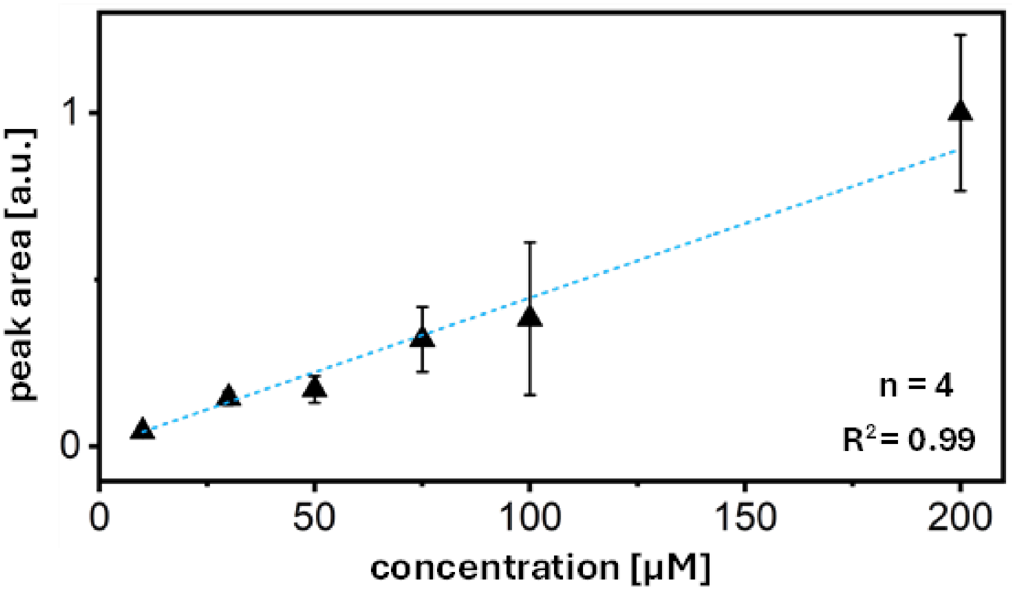

Figure S11 – Calibration curve for the quantification of α-tocopherol – Sample: 4 nL of 10, 30, 50, 75, 100 and 200 µM α-tocopherol dissolved in MeOH; mobile phase: 98:2 v/v CO2:MeOH (0.1% FA); column: 8.6 cm, silica (d_p = 3µm), T = 55 °C, $p_{precolumn}$ = 128 bar, $p_{postcolumn}$ = 102 bar; sheath flow: 15 µL/min; MS: 2.3 Hz, positive mode, −5.5 kV. The calculation of the tocopherol content in the potato chips was based on measurements of the extract (n = 5) conducted under identical conditions. The LOD values of the tocopherols (LOD_α: 2.5 µM, LOD_β: 10 µM, LOD_γ: 10 µM, LOD_δ: 30 µM) were calculated based on device-specific measurements.
